# ScRNA-seq reveals the correlation between M2 phenotype of tumor-associated macrophages and lymph node metastasis of breast cancer

**DOI:** 10.32604/or.2023.029638

**Published:** 2023-09-15

**Authors:** JUN SHEN, HONGFANG MA, YONGXIA CHEN, JIANGUO SHEN

**Affiliations:** 1 Department of Surgical Oncology, Sir Run Run Shaw Hospital, Zhejiang University School of Medicine, Hangzhou, China; 2 Department of Plastic Surgery, Sir Run Run Shaw Hospital, Zhejiang University School of Medicine, Hangzhou, China; 3 Laboratory of Cancer Biology, Sir Run Run Shaw Hospital, Zhejiang University School of Medicine, Hangzhou, China

**Keywords:** M2 macrophages, Breast cancer, Lymph node, Metastasis, ScRNA-seq

## Abstract

The process of lymphatic metastasis was proved to be associated with podoplanin-expressing macrophages in breast cancer (BC). This study aimed to investigate the role of the M2 phenotype of tumor-associated macrophages and mine the key M2 macrophages-related genes for lymph node metastasis in BC. We downloaded the GSE158399 dataset from the Gene Expression Omnibus (GEO) database, which includes transcriptomic profiles of individual cells from primary tumors, negative lymph nodes (NLNs), and positive lymph nodes (PLNs) of breast cancer patients. The cell subsets were identified by clustering analysis after quality control of the scRNA-seq using Seurat. The activation and migration capability of M2 macrophages were evaluated with R package “GSVA”. The key M2 macrophages-related genes were screened from the differential expressed genes (DEGs) and M2 macrophages activation and migration gene sets collected from MSigDB database. Our analysis identified three main cell types in primary tumors, NLNs, and PLNs: basal cells, luminal cells, and immune cell subsets. The further cell type classification of immune cell subsets indicated M2 macrophages accumulation in NLs and PLs. The GSVA enrichment scores for activation and migration capability were increased significantly in M2 macrophages from primary tumors than NLNs and PLNs (*p*-value < 0.001). Seven M2 macrophages activation-related and 15 M2 macrophages migration-related genes were significantly up-regulated in primary tumors than NLNs and PLNs. The proportion and GSVA enrichment scores for activation and migration of M2 macrophages may be potential markers for lymph node metastasis in breast cancer. Our study demonstrated that twenty-two up-regulated mRNA may be possible therapeutic targets for lymph node metastasis in breast cancer.

## Introduction

Breast cancer (BC) is the most frequently diagnosed neoplasm and the leading cause of cancer mortality among women [1]. In 2022, there will be estimated 290,560 new cases and 42,780 cancer deaths due to BC in the USA [2]. BC represents the most important cancer-related cause of disease burden worldwide, especially in developed countries [3]. Despite the continuous improvement of BC treatments in recent years, the survival of advanced BC is still not ideal. Lymph node metastasis is one of the main pathways of tumor metastasis, especially for breast tissue with abundant lymphatic vessels and lymphatic network [4]. The metastatic cancer cells in the lymph nodes can directly enter other lymphatic vessels and then spread widely in the body [5]. Lymph node metastasis is of great significance to BC’s prognosis, indicating the worsened prognosis [6]. Lymph node metastases is a complex biological process affected by a complex gene regulatory network and various growth factors, involving tumor movement, vascular invasion, and clonogenicity in the microenvironment [7]. In the process of lymph node metastases, lymph nodes provide a supportive environment for tumor cells of a specific genetic background, supporting their clonal growth and further distant metastasis [8].

The process of lymphatic metastasis in breast cancer has been found to be associated with a specific type of macrophages expressing podoplanin [9]. Macrophages display two different phenotypes in response to different environmental stimuli: M1 and M2 macrophages. M2 macrophages can produce large amounts of cytokines that cause Th2-type immune responses, suppressing immune function in the tumor microenvironment, inducing angiogenesis, and supporting tumor growth and metastasis [10]. In the tumor microenvironment, the majority of tumor-associated macrophages (TAMs) are of the M2 phenotype [11]. Increased numbers of M2 macrophages have been significantly correlated with lymph node metastasis, larger tumor size, poor differentiation of cancer cells, and an elevated risk of recurrence in BC [12]. The experimental data of Watari et al. showed that M2 macrophages correlated with lymph node metastasis in highly metastatic cancer [13]. Therefore, it is crucial to further investigate the biological characteristics of M2 macrophages in the context of breast cancer lymph node metastasis. Understanding their role and mechanisms in promoting metastasis could potentially lead to the development of targeted therapies.

With the development of sequencing technology, single-cell RNA sequencing (scRNA-seq) has brought new data at the cellular level to researchers, enriching the tools for single-cell analysis of tumors. The scRNA-seq enables researchers to explore the different biological properties of individual cells in complex tissues and understand the response of cell subpopulations to environmental elements. It has provided new solutions to various omics-related problems in the life sciences, and the related research has become increasingly popular. In this study, we collected a set of single-cell sequencing data (GSE158399) to investigate the role of M2 phenotype of tumor-associated macrophages and mine the key M2 macrophages-related genes for lymph node metastasis in breast cancer.

## Materials and Methods

### Data preparation and quality control

The single-cell sequencing data GSE158399 of lymph node metastasis in breast cancer was obtained from the GEO database (https://www.ncbi.nlm.nih.gov/geo/query/acc.cgi?acc=GSE158399). The paired single-cell data of three sources in a female patient with luminal B subtype were detected in the sequencing platform of HiSeq X Ten, including primary tumors (GSM4798908), NLNs (GSM4798910), and PLNs (GSM4798909). Table 1 shows the detailed cell and gene number statistics.

**Table 1:** Statistics of cell and gene numbers in the GSE158399 dataset before and after quality control

Cell number/Gene number	GSM4798908	GSM4798909	GSM4798910
Raw data	11467/32148	11356/29607	11323/29861
After QC	11338/16843	11307/13775	11212/12675

The number of detected cells and captured genes in the primary tumors, NLNs, and PLNs were counted. The intersection of the detected genes in the three tissues was collected, and the number of genes in every single cell of each sample was calculated. The threshold for quality control was determined by the overall distribution. The single cell and genes with low quality were eliminated using. “merge.SCTAssay” function in the R package “Seurat”.

### ScRNA-seq clustering analysis

To reduce genomic instability caused by the single cell quality, batch effect and retain most of the gene expression information, the R package “Seurat” was used to perform a series of data processing and analysis on the expression data of single cells. Based on this R package, we performed PCA dimensionality reduction, high-variant gene selection, and K-Nearest Neighbor clustering, and finally divided single cells into more detailed cell subsets and analyzed them.

### GSVA-gene set enrichment analysis

Relevant gene sets were collected through the MSigDB (https://www.gsea-msigdb.org/gsea/msigdb) database, and the R package “GSVA” was used to score the functional activity of M2 macrophages from the three samples using the Wilcoxon rank sum test compares differences among them.

### Screening and functional annotation of differentially expressed genes (DEGs)

The mean expression of each gene in the three samples was counted and the FC value was calculated. Subsequently, the Wilcoxon rank-sum test was performed on the single cells from the two types of samples, and the corresponding test *p*-value and the *p*-value corrected by the FDR algorithm were recorded, and genes with FDR < 0.001 and |log2FC| > 1 were retained. Finally, the DAVID (https://david-d.ncifcrf.gov/) database was used to analyze the KEGG signaling pathway enrichment of the above differentially expressed genes to understand the biological processes, signaling pathways, molecular functions, and cellular localization. The number of genes > 10 and *p* ≤ 0.05 were considered statistically significant.

## Results

### Data quality control results

**Figure 1:**
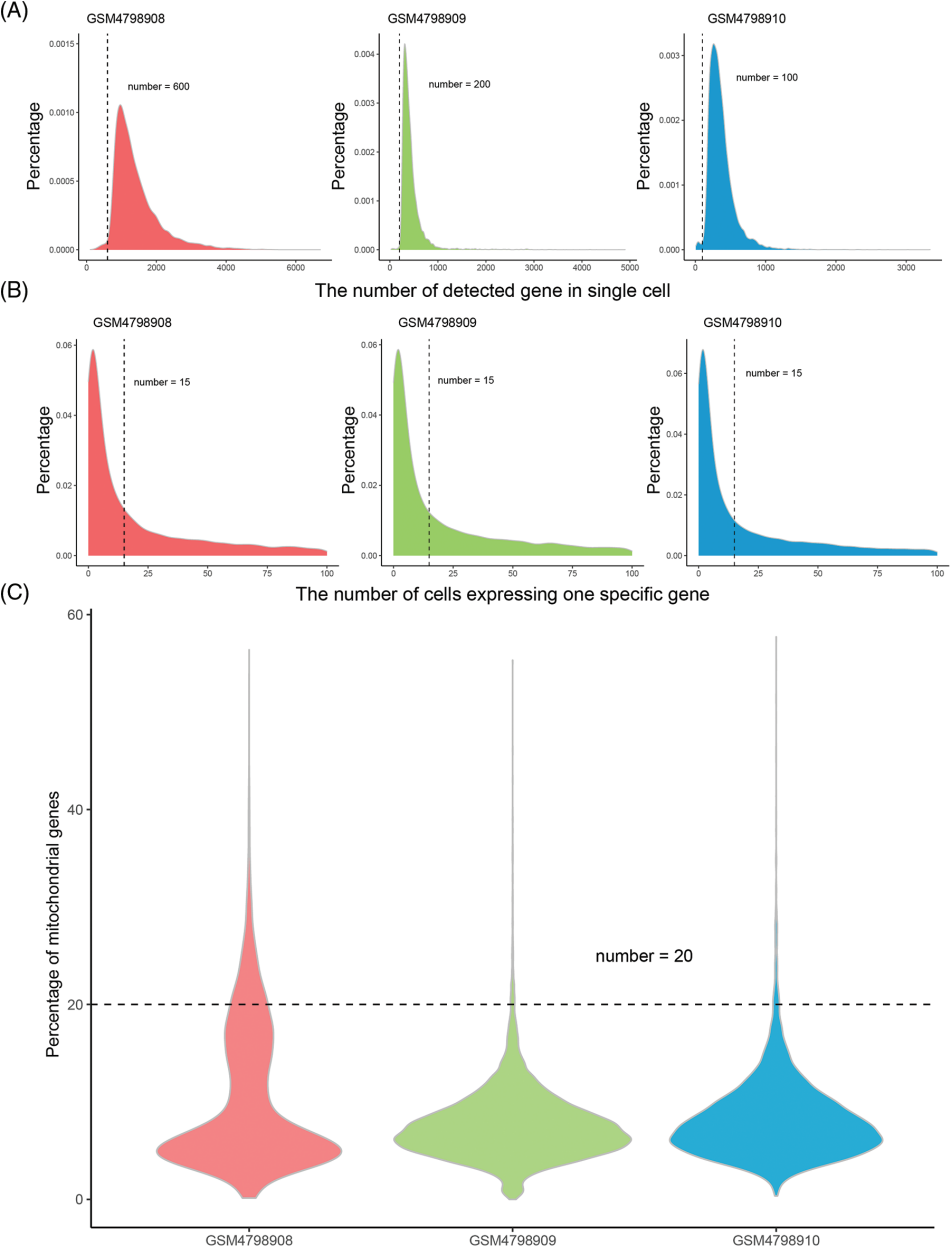
Distribution of gene numbers captured from single-cell.

High-quality, usable single-cell data were obtained after quality control processing. 24235 common genes were finally obtained after the intersection of the captured genes in the three tissues (Table 1). According to the overall distribution of the number of captured genes in single cells of the three samples, it is considered that the number of genes captured by cells in GSM4798908/GSM4798909/GSM4798910 is less than 600/200/100 genes are lower quality cells (Fig. 1A). Genes expressed in fewer than 15 cells were considered lower-quality genes based on the overall distribution of cell numbers expressed in each gene (Fig. 1B). After the above screening, 33857 single cell expression data were obtained by using the “merge. SCTAssay” function in the R package “Seurat”. Through the overall distribution of single cells from the three source samples, the proportion of mitochondrial genes in the integrated single-cell data was counted, and cells with mitochondrial gene expression accounting for more than 20% were excluded [14]. Ultimately, 17,746 high-quality genes and 32,079 single-cell samples were obtained (Fig. 1C).

### Major cell types in different tissues

**Figure 2:**
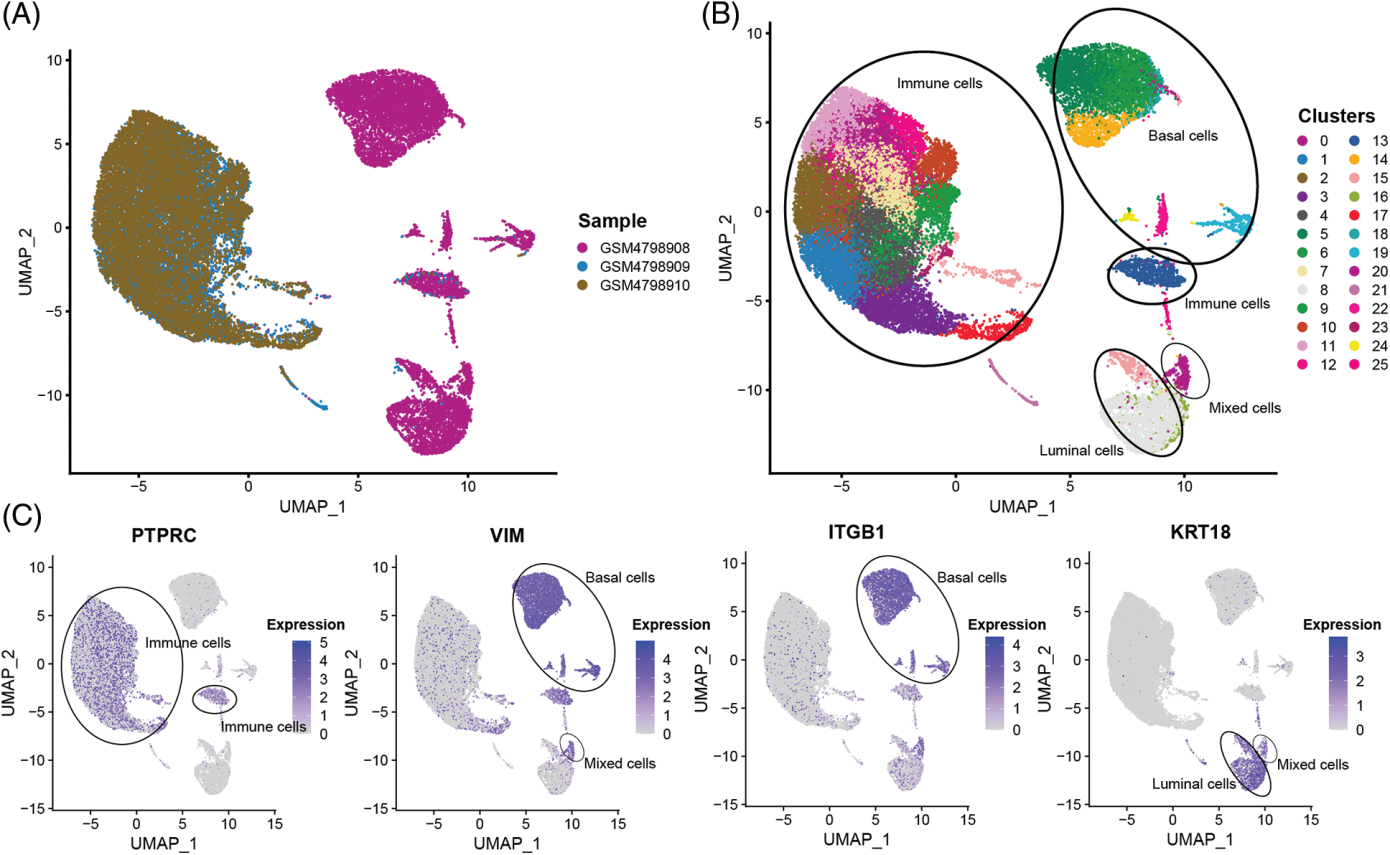
The sample source and cell subgroups in scRNA-seq data after clustering.

In order to obtain a more detailed analysis of single cells, The 32,079 single cells were divided into 26 cell subsets using the R package “Seurat”. By comparing the tissue sources of the single-cell samples, it was found that the single cells in the GSM4798909 and GSM4798910 samples were generally immune leukocytes. In contrast, a small number of cells in the primary tumor GSM4798908 were immune leukocytes (Figs. 2A and 2B). On this basis, three main cell types present in breast tissue were identified according to gene markers expressed explicitly in cell subsets: Basal cells (VIM^+^, ITGB1/CD29^+^), Luminal cells (KRT19^+^), and immune cells Cell subsets (PTPRC/CD45^+^) [15–17]. In addition, a small group of mixed cells expressed both Basal cells and Luminal cell markers (Figs. 2B and 2C). Since KRT14 and KRT5 were not detected in the dataset, they could not be used as identification criteria for Basal cells.

### M2 macrophages accumulate in PLNs

**Figure 3:**
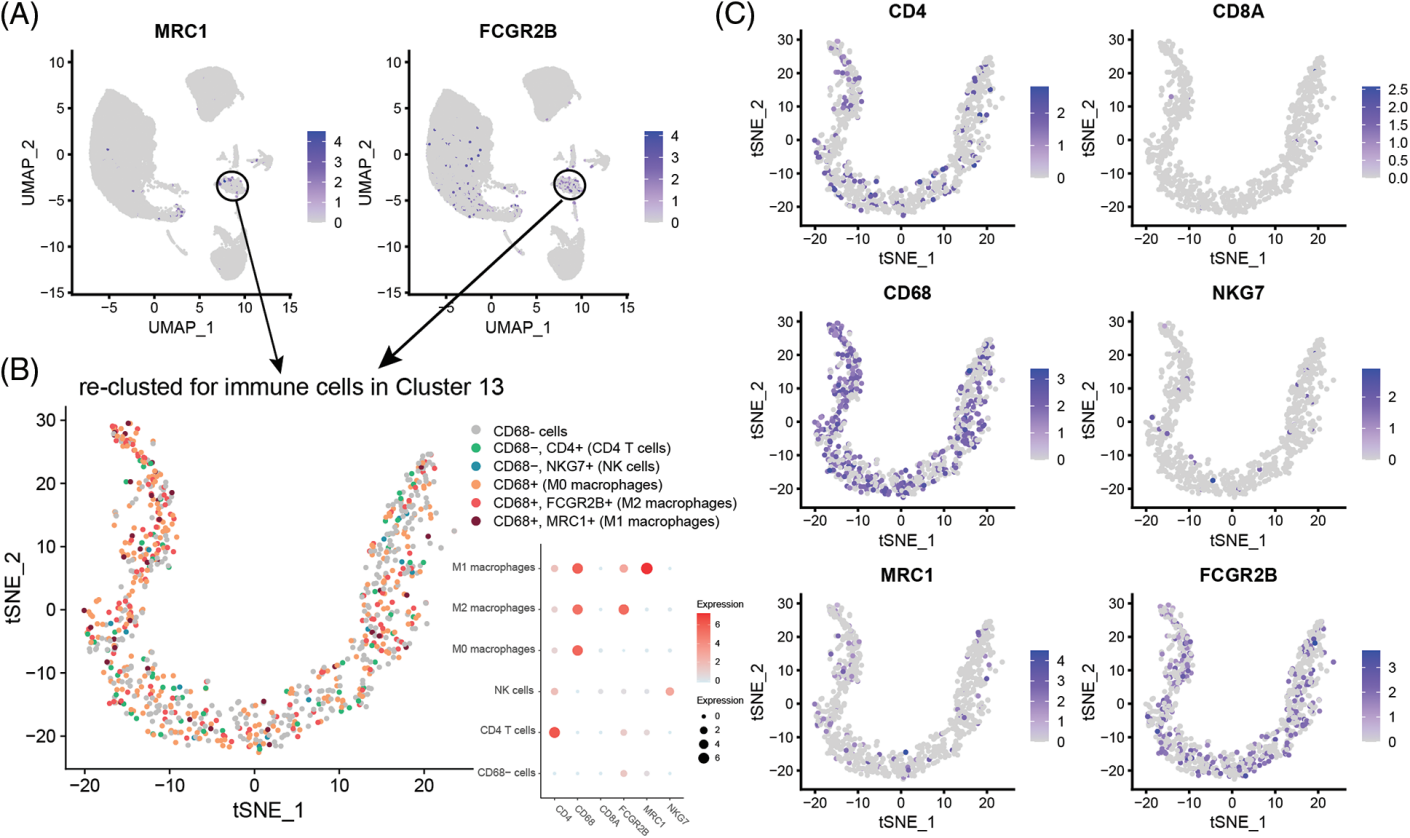
Macrophage distribution in single cells from three sample sources.

Cell type division of the immune cell subset Cluster 13 present in single cells derived from the GSM4798908 sample. The results showed that there were a small number of T cells (62) and NK cells (14) in this cell population (850 cells in Cluster 13). Still, most of them were macrophages that specifically expressed CD68, especially M2 macrophages that specifically expressed FCGR2B, and a few were M1 macrophages specifically expressing MRC1 (Figs. 3A and 3B). Second, M2-type macrophages were significantly more numerous than M1-type in GSM4798909 and GSM4798910 tissues (Fig. 3C). In lymph node metastasis positive tissue GSM4798909-derived single cells, the number of M2-type macrophages was 6.7708 times that of M1-type macrophages. In single cells derived from GSM4798908 and GSM4798910, the number of M2 macrophages is 3.4416 and 5.8000 times that of M1 macrophages, respectively (Table 2).

**Table 2:** The proportion of macrophages in the three samples

Sample	M1 macrophage/leukocytes	M2 macrophage/leukocytes	M2/M1
GSM4798908 (cluster 13)	77/850	265/850	3.44
GSM4798909	48/11165	325/11165	6.77
GSM4798910	50/10991	290/10991	5.80

### The ability of M2 macrophages to migrate and activate is significantly enhanced in orthotopic tumor tissue

**Figure 4:**
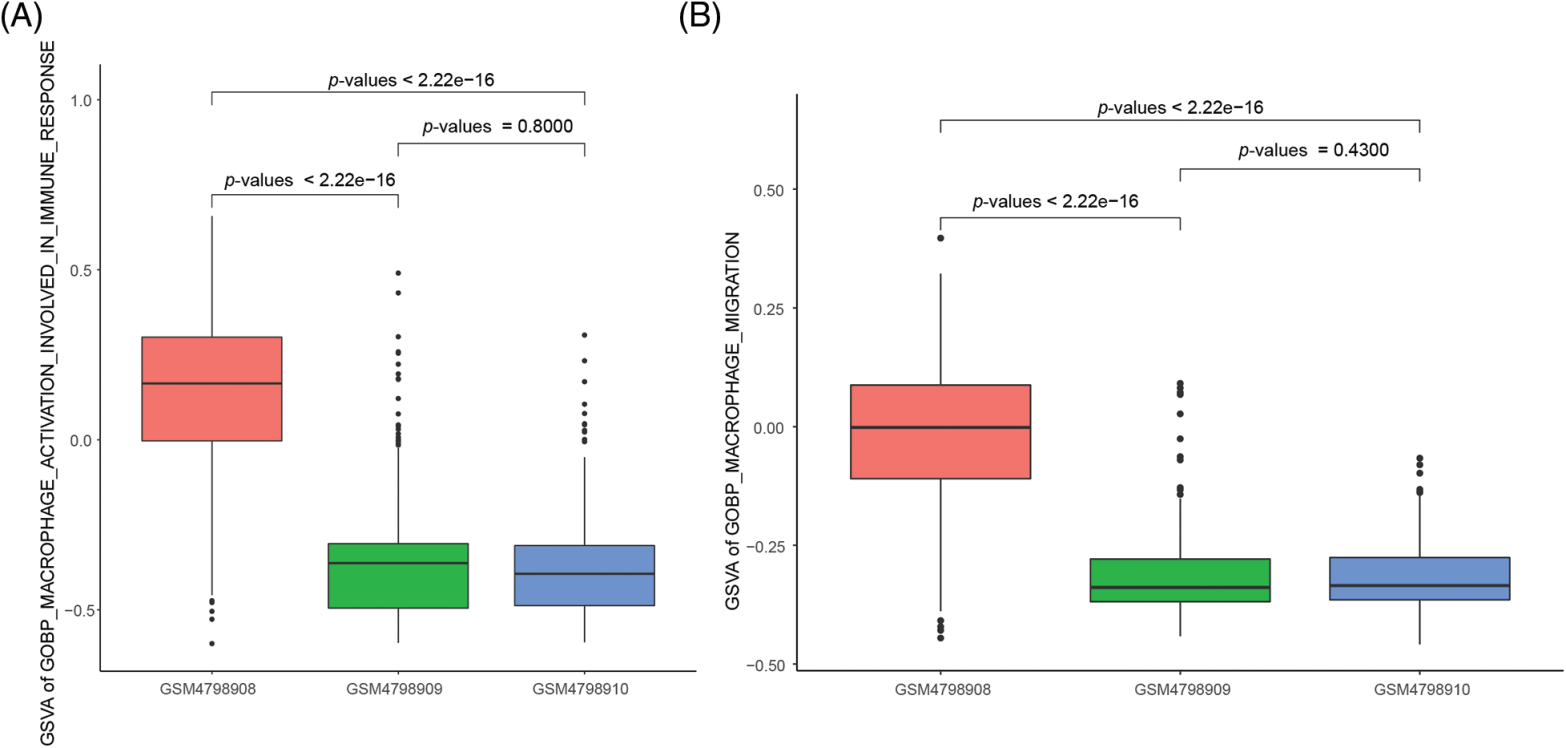
Differences in the activation capacity and cell migration ability of single-cell M2 macrophages.

For this study, we focused on the ability of M2 macrophages to migrate and activate cellular immune responses. For this study, we focused on the ability of M2 macrophages to migrate and activate cellular immune responses. We used the “GOBP_MACROPHAGE_ACTIVATION_INVOLVED_ IN_IMMUNE_RESPONSE” gene set from the MSigDB database to assess macrophage activation, and the “GOBP_MACROPHAGE_MIGRATION” gene set to evaluate macrophage migration. The results demonstrated that M2 macrophages in orthotopic tumor tissue exhibited significantly enhanced activation ability compared to M2 macrophages in NLNs and PLNs. The statistical analysis yielded test *p*-values of less than 2.2e-16, indicating a highly significant difference (Fig. 4A). Furthermore, the migration ability of M2 macrophages in orthotopic tumor tissue was found to be significantly higher than that of M2 macrophages in NLNs and PLNs. The statistical analysis resulted in a test *p*-value of less than 2.2e-16, indicating a highly significant difference (Fig. 4B). These findings suggest that M2 macrophages in orthotopic tumor tissue possess a greater propensity to migrate and activate cellular immune responses compared to M2 macrophages in the nearby and peripheral lymph nodes. The enhanced migratory and activation abilities of M2 macrophages in the tumor microenvironment may contribute to the regulation of immune responses and potentially influence tumor progression and immune surveillance.

### M2 macrophages in orthotopic tumors promote tumor cell metastasis through phagosomes and immunodeficiency

**Figure 5:**
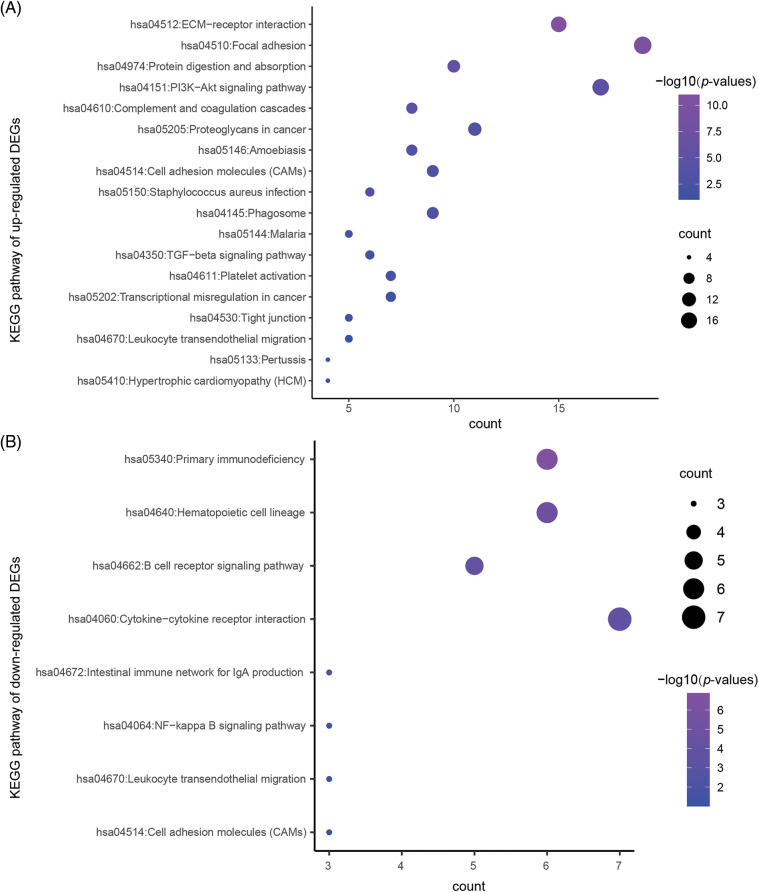
The KEGG pathway enriched in the significantly DEGs.

To reveal the specific role of M2 macrophages in lymph node metastasis, we obtained 265, 325, and 290 single cells from GSM4798908/GSM4798909/GSM4798910, respectively. Finally, 1854 significantly differentially expressed genes were selected, including 1755 significantly overexpressed genes, and 99 significantly underexpressed genes. The top 200 highly expressed genes were functionally enriched using the DAVID tool. It was found that the significantly overexpressed genes were related to the following related KEGG pathways: “hsa04512:ECM-receptor interaction”, “hsa04151:PI3K-Akt signaling pathway”, “hsa05205:Proteoglycans in cancer”, “hsa04145:Phagosome”. In addition, the top 200 genes with significantly high expression were also involved in “hsa04510:Focal adhesion”, “hsa04974:Protein digestion and absorption”, “hsa04610:Complement and coagulation cascades”, “hsa05146:Amoebiasis”, “hsa04514:Cell adhesion molecules (CAMs)”, “hsa05150:*Staphylococcus aureus* infection”, etc. (Table 3). The GO enrichment bubble chart was drawn according to the top 18 GO BP terms involved in the above genes (Fig. 5A).

**Table 3:** The KEGG pathway enriched in the top 200 significantly highly expressed genes

Term	Count	*p*-value
hsa04512:ECM-receptor interaction	15	9.47E-12
hsa04510:Focal adhesion	19	3.12E-10
hsa04974:Protein digestion and absorption	10	3.17E-06
hsa04151:PI3K-Akt signaling pathway	17	1.90E-05
hsa04610:Complement and coagulation cascades	8	4.41E-05
hsa05205:Proteoglycans in cancer	11	4.41E-04
hsa05146:Amoebiasis	8	6.54E-04
hsa04514:Cell adhesion molecules (CAMs)	9	7.77E-04
hsa05150:*Staphylococcus aureus* infection	6	8.78E-04
hsa04145:Phagosome	9	0.0011
hsa05144:Malaria	5	0.0046
hsa04350:TGF-beta signaling pathway	6	0.0062
hsa04611:Platelet activation	7	0.0094
hsa05202:Transcriptional misregulation in cancer	7	0.02883
hsa04530:Tight junction	5	0.03291
hsa04670:Leukocyte transendothelial migration	5	0.0765
hsa05133:Pertussis	4	0.0868
hsa05410:Hypertrophic cardiomyopathy (HCM)	4	0.0949

Similarly, 99 genes that were significantly downregulated were also functionally annotated, and it was found that the down-regulated genes were mainly involved in the following related KEGG pathways: “hsa05340:Primary immunodeficiency”, “hsa04662:B cell receptor signaling pathway”, “hsa04672:Intestinal immune network” for IgA production. In addition, the down-regulated 99 genes are also involved in “hsa04640:Hematopoietic cell lineage”, “hsa04060:Cytokine-cytokine receptor interaction”, “hsa04064:NF-kappa B signaling pathway”, “hsa04670:Leukocyte transendothelial migration”, “hsa04514:Cell adhesion molecules (CAMs)” and other signaling pathways (Table 4, Fig. 5B).

**Table 4:** 99 significantly down-regulated genes involved in the KEGG pathway

Term	Count	*p*-value
hsa05340:Primary immunodeficiency	6	1.33E-07
hsa04640:Hematopoietic cell lineage	6	1.54E-05
hsa04662:B cell receptor signaling pathway	5	1.17E-04
hsa04060:Cytokine-cytokine receptor interaction	7	2.32E-04
hsa04672:Intestinal immune network for IgA production	3	0.0133
hsa04064:NF-kappa B signaling pathway	3	0.0422
hsa04670:Leukocyte transendothelial migration	3	0.0694
hsa04514:Cell adhesion molecules (CAMs)	3	0.0997

### Differential analysis of key genes related to M2 macrophages

**Figure 6:**
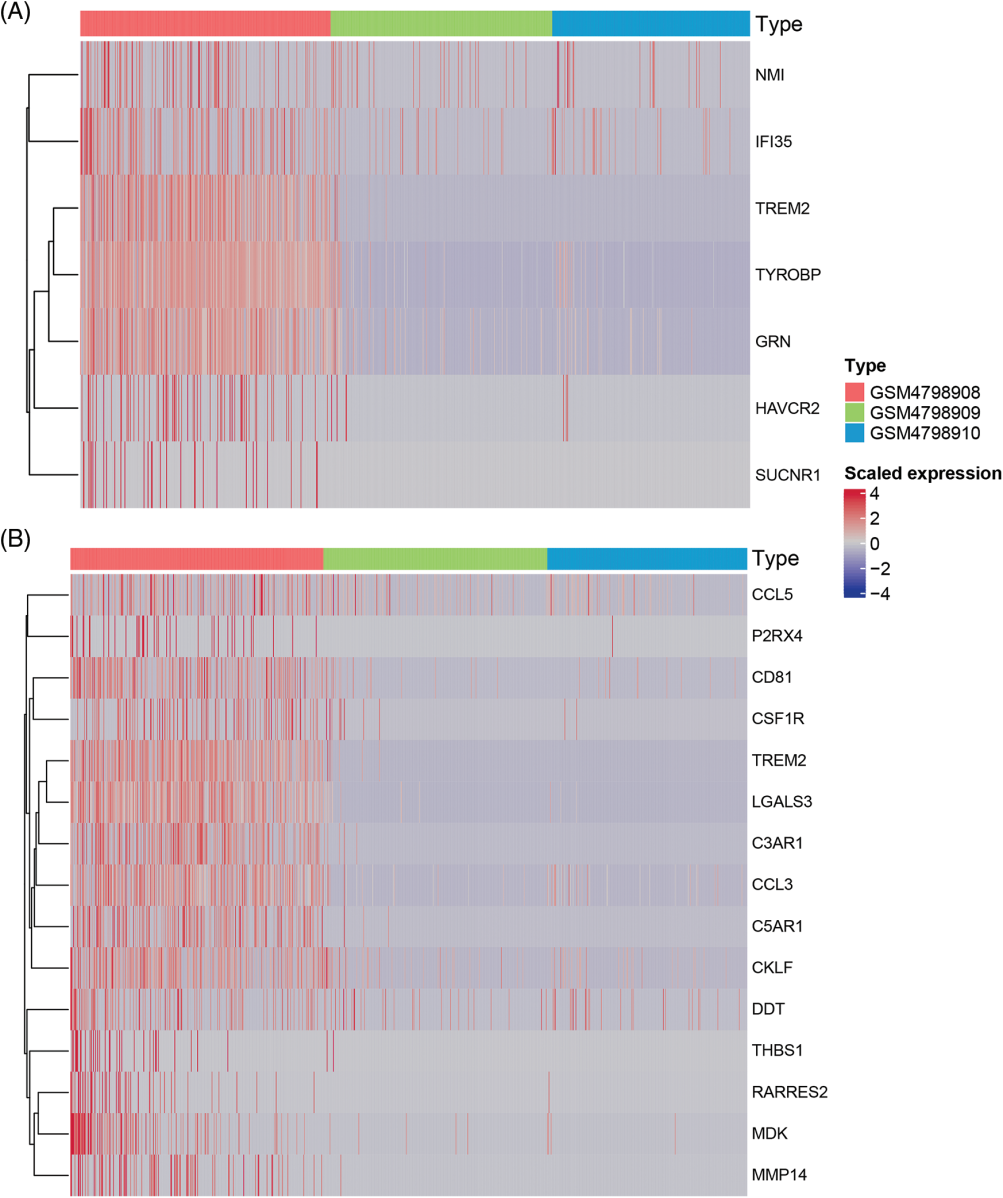
Heatmap of macrophage immune responses and migration related DEGs.

According to the above signal pathway results, the promotion of lymph node metastasis by M2 macrophages may be related to biological characteristics such as phagosomes and immunodeficiency. Therefore, through the statistics of relevant genes in “gobp_macrophage_activation_invoved_ in_immune_response”, we found that there are seven significantly up-regulated genes, namely SUCNR1 (Succinate Receptor 1), TREM2 (Triggering Receptor Expressed On Myeloid Cells 2), TYROBP (Tyrosine Kinase Binding Protein), GRN (Granulin Precursor), HAVCR2 (Hepatitis A Virus Cellular Receptor 2), IFI35 (Interferon Induced Protein 35), NMI (N-Myc And STAT Interactor). In contrast, the SUCNR1 gene was not expressed in the two lymph node-derived samples, The TREM2 gene was not expressed in lymph node metastasis-negative samples (Table 5). To visualize the differential expression of these seven upregulated genes, a heat map was generated (Fig. 6A).

**Table 5:** Differential expression statistics of activation-related genes in seven macrophage immune responses

Gene	Mean expression in GSM4798910	Mean expression in GSM4798909	Mean expression in GSM4798908	FC (GSM4798908 *vs*. GSM4798910)	wilcox.test *p*-value (GSM4798908 *vs*. GSM4798910)	FC (GSM4798908 *vs*.GSM4798909)	wilcox.test *p*-value (GSM4798908 *vs*. GSM4798909)
SUCNR1	0.0000	0.0000	0.1035	Inf	4.09E-07	Inf	8.53E-08
TREM2	0.0000	0.0394	1.3976	Inf	3.51E-75	35.4874	6.60E-77
TYROBP	0.1511	0.2219	3.1569	20.8919	1.45E-100	14.2280	5.20E-101
GRN	0.0819	0.1783	1.4668	17.9068	4.16E-77	8.2265	2.97E-71
HAVCR2	0.0103	0.0159	0.1742	16.8376	3.62E-11	10.9497	1.00E-11
IFI35	0.0992	0.0731	0.4317	4.3539	1.92E-15	5.9064	1.05E-19
NMI	0.0647	0.0769	0.2398	3.7085	5.76E-08	3.1179	7.32E-07

Similarly, we performed differential expression statistics on the “GOBP_MACROPHAGE_MIGRATION” gene set, revealing 15 genes that were significantly up-regulated. These genes included C3AR1 (complement component 3a receptor1), C5AR1 (complement component 5a receptor1), MMP-14 (Matrix metalloproteinase-14), THBS1 (thrombospondin-1), TREM2 (triggering receptor expressed on myeloid cells 2), LGALS3 (Galectin 3), RARRES2 (Retinoic Acid Receptor Responder 2), CSF1R (Colony Stimulating Factor 1 Receptor), P2RX4 (Purinergic Receptor P2X 4), MDK (Midkine), CD81 (CD81 Molecule), CCL3 (C-C Motif Chemokine Ligand 3), CKLF (Chemokine Like Factor), CCL5 (C-C Motif Chemokine Ligand 5), DDT (D-Dopachrome Tautomerase).

In particular, C3aR1, C5aR1, MMP-14, THBS1, and TREM2 were abnormally highly expressed in orthotopic tumor tissue but were not expressed in lymph node metastasis-negative tissues (Table 6). To visualize these up-regulated genes, we generated a differentially expressed heatmap, highlighting their expression patterns (Figs. 6A and 6B).

**Table 6:** Differential expression statistics of 15 macrophage migration-related genes

Gene	Mean expression in GSM4798910	Mean expression in GSM4798909	Mean expression in GSM4798908	FC (GSM4798908 *vs*. GSM4798910)	wilcox.test *p*-value (GSM4798908 *vs*. GSM4798910)	FC (GSM4798908 *vs*. GSM4798909)	wilcox.test *p*-value (GSM4798908 *vs*. GSM4798909)
C3AR1	0.0000	0.0172	0.6209	Inf	2.08E-43	36.1320	2.67E-44
C5AR1	0.0000	0.0141	0.5454	Inf	8.42E-34	38.6632	4.49E-35
MMP14	0.0000	0.0031	0.2432	Inf	2.53E-15	79.0505	2.90E-16
THBS1	0.0000	0.0062	0.1070	Inf	2.61E-07	17.3856	8.30E-07
TREM2	0.0000	0.0394	1.3976	Inf	3.51E-75	35.4874	6.60E-77
LGALS3	0.0138	0.0490	1.7323	125.5901	2.55E-83	35.3530	4.19E-86
RARRES2	0.0034	0.0000	0.1657	48.0558	7.01E-09	Inf	1.97E-10
CSF1R	0.0069	0.0387	0.2793	40.5018	1.49E-18	7.2264	1.33E-16
P2RX4	0.0034	0.0000	0.1083	31.4139	7.14E-08	Inf	2.50E-09
MDK	0.0138	0.0154	0.3830	27.7650	6.38E-18	24.8927	2.17E-19
CD81	0.0365	0.0639	0.6914	18.9438	3.50E-36	10.8273	1.84E-35
CCL3	0.1812	0.0932	1.8583	10.2552	1.02E-53	19.9381	1.03E-66
CKLF	0.0992	0.1567	0.8371	8.4426	1.43E-37	5.3415	7.84E-35
CCL5	0.2341	0.2977	0.7871	3.3615	8.75E-12	2.6435	5.96E-10
DDT	0.0936	0.0974	0.2939	3.1393	2.17E-08	3.0165	1.37E-08

## Discussion

Lymph node metastasis is a common problem in breast cancer, which seriously affects the survival and prognosis of patients. Therefore, it has become increasingly urgent to understand its pathogenesis and find a specific and sensitive treatment method. The mobility and invasiveness of cells are the keys to tumor metastasis, and the movement and migration of tumor cells play a leading role in the whole process of tumor metastasis. The study conducted by Watari et al. [18] stated that inflammatory stimuli could help to establish the tumor microenvironment, and the growth, invasion, and matastases could be induced through the activation of macrophages. In our study, we observed a fundamental distinction between samples obtained from primary tumors and lymph nodes. The single-cell analysis of lymph node samples revealed a predominant presence of immune leukocytes, whereas immune leukocytes constituted only a small portion of the primary tumor samples. Further classification of cell types based on single-cell data demonstrated that the majority of cells in the primary tumors were macrophages expressing the CD68 marker, particularly M2 macrophages expressing FCGR2B. Additionally, Mahmoud et al. [19] discovered that higher numbers of CD68+ macrophages were associated with lower overall survival rates in breast cancer patients. This finding underscores the potential significance of M2 macrophages in lymph node metastasis and its clinical implications.

Tumor-associated macrophages (TAMs) play a key role in the growth of breast cancer. The role of TAMs in cancer pathogenesis depends on their phenotypic and functional polarization [20]. Tumor cells can disguise as normal cells to deceive macrophages and inhibit the phagocytosis of tumor cells by macrophages [21]. Studies have shown that the high infiltration of TAMs is essential in promoting breast cancer cell metastasis. The infiltration density of TAMs in primary breast tumor tissue is significantly higher than in adjacent tissue. At the same time, the infiltration of TAMs in primary breast tumor tissue increases with tumor stage and size [22]. Previous studies have shown that infiltration of macrophages in the tumor microenvironment supported tumor growth, angiogenesis, metastasis/invasion, inflammation, and immunosuppression by secreting factors that promote tumor progression [23]. TAMs are heterogeneous populations with different subsets performing different functions [24]. TAMs primarily exhibit a phenotype resembling alternatively activated M2 macrophages, which possess anti-inflammatory properties and promote tumor growth. M2-type macrophages largely contribute to the aggressiveness of malignant tumors [18]. TAMs located in primary tumors are mostly associated with poor prognosis in cancer patients. In this study, we found that M2 macrophages mainly aggregated in breast cancer lymph node metastasis-positive samples, and M2 macrophages in orthotopic tumor tissue had stronger migration and activation ability. This phenomenon suggests to us that M2 macrophages play an important role in promoting lymph node metastasis in patients. Depletion or phenotypic reversal (M2 to M1) of TAMs has been shown to halt tumor progression in mouse models of breast cancer [25]. In order to reveal the biological role of M2 macrophages in the process of lymph node metastasis, we analyzed the differences of M2 macrophages from three samples and annotated their functions. The results showed that M2 macrophages may help tumor cells complete metastasis through phagosomes, and M2 macrophages may also have immune function defects. New clinical research suggested that an immunodeficiency of macrophages 22 may cause Crohn’s disease (CD). The reason for granulomas is the reduced ability to clear invading bacteria induced by a weakened attraction of granulocytes to the intestinal wall. So far, there are few reports on whether M2 macrophages promote tumor cell metastasis through phagosomes and immunodeficiency.

Based on the above analysis, we analyzed the difference between the gene sets of “gobp_macrophage_activation_ involvedin_immune_response” and “gobp_macrophage_ migration” in M2 macrophages, respectively. The previous study analyzed the data in GSE158399 and found the elevated expression level of CXCL14 in PLs, which might be valuable in predicting the prognosis of breast cancer with lymph node metastasis [23]. In this study, we found that SUCNR1, C3aR1, C5aR1, MMP14, THBS 1, TSP-1, and TREM2, which are abnormally expressed in tumor tissue *in situ*, may be potentially associated with lymph node metastasis.

Keiran et al. [26] discovered that the activation of SUCNR1 plays a crucial role in promoting the anti-inflammatory phenotype of macrophages, thus contributing to the anti-inflammatory response. It is demonstrated that Succinate and its receptor SUCNR1 can suppress immune responses. Moreover, the receptor SUCNR1 can also suppresse immune responses, and SUCNR1 deficiency in macrophages could lead to enhanced inflammatory responses [27]. C3AR1 was down-regulated in osteosarcoma tissues and cells, and its overexpression inhibited the proliferation, migration, and invasion of osteosarcoma cells and induced apoptosis [28]. These findings may appear contradictory to our conclusions, but they emphasize that the interaction between C3AR1 and cell migration can have divergent roles in different types of cancer. In addition, C5α can also promote the malignant development of HBc-positive hepatocellular carcinoma through C5AR1 [29]. Matrix metalloproteinases (MMPs) are key factors in extracellular matrix remodeling and cell migration during tumor metastasis. In particular, MMP-14, a membrane-anchored MMP, is closely involved in these processes. The study found that Bladers I and IV were associated with cell migration. Bladers IV is required for MMP-14 homodimerization. The interaction between MMP-14 and CD44 leads to phosphorylation of the EGF receptor and downstream activation of MAPK and PI3K signaling pathways involved in cell migration [30]. Reports provide evidence [31] that THBS1 derived from oral squamous cell carcinoma (OSCC) exosomes is involved in the polarization of macrophages towards an M1-like phenotype.

In contrast, conditioned medium from exosomes induced M1-like TAMs and significantly promoted the malignant migration of OSCCs. Perturbation of macrophage migration inhibitory factor expression in mouse melanoma suppresses tumor formation by up-regulating Thrombospondin-1 (TSP-1) [32]. TSP-1 is a secreted protein that inhibits angiogenesis, modulates anti-tumor immunity, stimulates tumor cell migration, and modulates extracellular proteases and growth factors in the tumor microenvironment. Furthermore, in polyomavirus middle T antigen (Pyt) transgenic mice, TSP-1 in the mammary tumor microenvironment inhibits angiogenesis and tumor growth, yet promotes lung metastasis in Pyt transgenic mice [33]. Research has shown that targeting TREM2 on tumor-associated macrophages enhances immunotherapy [34].

However, there are potential limitations in this study. Firstly, the primary breast cancer tissues, PLs, and NLs in the GSE158399 dataset were from one patient with Luminal B subtype breast cancer, which may restrict the generalizability of the findings. Secondly, the current mainstream tissue sequencing methods may not effectively detect the key genes associated with M2 macrophages. Therefore, there could be additional genes involved in the M2 macrophage phenotype that were not identified in this study. Lastly, further investigations are needed to validate and expand upon the interesting findings presented here. In conclusion, this study found that M2 macrophages play an important role in promoting lymph node metastasis in breast cancer patients, possibly through immune activation and phagosomes to help tumor cells complete metastasis, thus exerting their biological function. In addition, we also discovered key genes that M2 macrophages significantly up-regulated in immune response and cell migration to discover key molecular targets regulating breast cancer metastasis and providing new strategies for the prevention and treatment of breast cancer metastasis. These results may have important implications for understanding the mechanism of lymph node metastasis in breast cancer.

## Data Availability

The single-cell sequencing data GSE158399 of lymph node metastasis in breast cancer was obtained from the GEO database (https://www.ncbi.nlm.nih.gov/geo/query/acc.cgi?acc=GSE158399).
